# Identification of Potential Gene Regulatory Pathways Affecting the Ratio of Four-Seed Pod in Soybean

**DOI:** 10.3389/fgene.2021.717770

**Published:** 2021-09-01

**Authors:** Ting Fang, Yiwei Bai, Wenxuan Huang, Yueying Wu, Zhihui Yuan, Xiaoyan Luan, Xinlei Liu, Lianjun Sun

**Affiliations:** ^1^State Key Laboratory of Agrobiotechnology, Beijing Key Laboratory for Crop Genetic Improvement and College of Agronomy and Biotechnology, China Agricultural University, Beijing, China; ^2^Institute of Soybean Research, Heilongjiang Provincial Academy of Agricultural Sciences, Harbin, China

**Keywords:** soybean, four-seed pod, bulked segregant RNA sequencing, differentially expressed gene, gene regulatory pathway

## Abstract

The number of four-seed pods is one of the most important agronomic traits affected by gene and environment that can potentially improve soybean (*Glycine max*) yield. However, the gene regulatory network that affects the ratio of four-seed pod (the ratio of the number of four-seed pods to the total number of pods in each individual plant) is yet unclear. Here, we performed bulked segregant RNA sequencing (BSR-seq) on a series of recombinant inbred lines (RILs) derived from hybrid progenies between Heinong 48 (HN48), a cultivar with a high ratio of four-seed pod, and Henong 64 (HN64), a cultivar with a low ratio of four-seed pod. Two tissues, flower bud and young pod, at two different growth stages, R1 and R3, were analyzed under the ratios of four-seed pod at less than 10% and greater than 30%, respectively. To identify the potential gene regulation pathways associated with the ratio of soybean four-seed pod, we performed differentially expressed analysis on the four bulked groups. A differentially expressed gene (DEG) encoding a photosystem II 5-kDa protein had the function of participating in the energy conversion of photosynthesis. In addition, 79 common DEGs were identified at different developmental stages and under different ratios of four-seed pod. Among them, four genes encoding calcium-binding proteins and a WRKY transcription factor were enriched in the plant–pathogen interaction pathway, and they showed a high level of expression in roots. Moreover, 10 DEGs were identified in the reported quantitative trait locus (QTL) interval of four-seed pod, and two of them were significantly enriched in the pentose and glucuronate interconversion pathway. These findings provide basic insights into the understanding of the underlying gene regulatory network affected by specific environment and lay the foundation for identifying the targets that affect the ratio of four-seed pod in soybean.

## Introduction

Soybean (*Glycine max* [L.] Merr.) is one of the most important protein and oil crops worldwide (Wang and Tian, 2015). The increase of soybean yield is not only affected by the number of pods per plant, the number of seeds per pod, and the weight of seeds but also restricted by the environment (Yang et al., 2013; Liu et al., 2015). The ratio of four-seed pod as a vital factor affecting yield trait is jointly regulated by genotype and environment (Liu et al., 2015). The influence of environment is potentially realized through the gene regulation network (Huang et al., 2020). Therefore, exploring the pathways of genes involved is the key to understanding the formation of four-seed pod under different four-seed pod ratios, which implies the phenotypic effect of gene and environment (Guo et al., 2020).

The number of four-seed pods, as an important agronomic trait, is a potential factor influencing yield, and it is also one of the critical goals for the selection and breeding of high-yield soybean accessions (Richards, 2000). The number of four-seed pods is a complex quantitative trait and is easily affected by environmental conditions (Tischner et al., 2003; Fang et al., 2013). The ratio of four-seed pod is usually low in cultivated soybean accessions. To improve the ratio of four-seed pod in soybean, it is essential to obtain excellent elite varieties and identify effective gene loci. At present, six stable main effect quantitative trait loci (QTLs) controlling the number of four-seed pods have been identified among 11 different environments (Asakura et al., 2012; Yang et al., 2013). Genetics studies have shown that only *Ln* gene in soybean has been cloned, which is considered to be one of the main genes that control the number of four-seed pods in soybean (Jeong et al., 2012). And *Ln* gene might affect the formation of pods by regulating cell division and cell expansion to control the formation of ovule (Fang et al., 2013; Sayama et al., 2017). These results laid the foundation for further research on the genes that affect the ratio of four-seed pod. Moreover, many minor effect QTLs affected by environment have also been identified (Gutierrez-Gonzalez et al., 2009; Funatsuki et al., 2012), and their roles in affecting the ratio of four-seed pod were rarely reported. Overall, *Ln* gene affects the external outline of the formation of four-seed pod, while the exploration of gene regulation pathways in specific environment is to understand the internal details of the formation of four-seed pod under different four-seed pod ratios. This implies the phenotypic effect of environment conditions, namely, environmental adaptability, and is also an important aspect of adaptive breeding.

The development of fruit pods was related to a variety of gene regulatory pathways, among which abscisic acid (ABA) and calcium signal pathways have been reported. A study has found that ABA secreted by the roots can control the expansion of the early pods during the critical stage of 3–5 days after flowering (Liu et al., 2004). The high level of ABA in the kernels or pods can affect the abortion of young ovaries by inhibiting cell division (Dembinska et al., 1992; Setter and Flannigan, 2001; Liu et al., 2003). Calcium is an essential nutrient element in the process of plant growth and development, which is mainly transported from the roots to aboveground leaves through the xylem (Zhang et al., 2021). A lack of calcium caused empty pods in peanut (Yang et al., 2020). Calcium has been shown to not only enhance the nutrients accumulation but also activate the hormone-related signaling pathway such as ABA signals in the development of plants (Yang et al., 2017; Zhang et al., 2021). Although root-derived ABA and calcium signaling regulatory pathways can be involved in the formation of crop pods, their influence on the ratio of soybean four-seed pod under natural conditions has not been reported yet.

Studying the factors affecting the ratio of four-seed pod plays a significant role in improving soybean yield (Fang et al., 2013; Liu et al., 2015). The gene regulatory pathways in specific environments often cause specific phenotypic effects (Xue et al., 2019). Therefore, in order to study how gene regulatory pathways affect the ratio of four-seed pod, we constructed a genetic population using two elite soybean cultivars HN48 and HN64 from the Heilongjiang province of China. Bulked segregant RNA sequencing (BSR-seq) was conducted on the progenies. Flowering period and young pod period are two key developmental stages for the formation of soybean pods. We chose flower buds and young pods as experimental materials. Differentially expressed genes (DEGs) were identified in four bulked groups; each bulked group consisted of the lines selected from the recombinant inbred line (RIL) with the ratio of four-seed pod of less than 10% and greater than 30% at the two different developmental stages. In this study, we obtained a DEG related to photosynthesis, which encodes a photosystem II protein. Four DEGs encoding calcium-binding proteins and a WRKY transcription factor were significantly enriched in plant–pathogen interaction pathway, which may affect the ratio of four-seed pod of the aboveground plants through the action of underground roots. Moreover, we identified two DEGs related to pectin, *Glyma.13G060900* and *Glyma.13G064700*, and they overlapped with a major QTL controlling the trait of four-seed pod number on chromosome 13. They may be important candidate genes that affect the ratio of four-seed pod. These results helped us to understand the genetic basis that affects the ratio of four-seed pod in soybean.

## Materials and Methods

### Plant Materials

To identify gene expression associated with four-seed pod of soybean, we generated a RIL population using single seed descendant method with at least five generations. The population was constructed using parent HN64 and HN48. The female parent HN64, is a major high-yielding cultivar with a low ratio of four-seed pods, while the male parent HN48, has a high ratio of four-seed pods, they were all from the Heilongjiang province. A total of 353 RILs were constructed and used in this study. The RILs were planted at Shangzhuang Experimental Station of China Agricultural University in the summer of 2019.

According to the developmental tissues of soybean, the flower buds (at R1 stage) and young pods (at R3 stage) were collected, quickly frozen in liquid nitrogen, and stored at −80°C for RNA extraction. We harvested the samples, counted the ratio of the number of four-seed pods to the total number of pods in each individual plant, and took the average percent of four individuals as the ratio of four-seed pod of each line. According to the proportion of four-seed pods of each line, the ratios of four-seed pod were divided into less than 10% and greater than 30% at two different levels. Each bulked group consisted of the typical lines that were selected from the RIL population according to the different ratios of four-seed pod. Each stage contained two bulked groups, and a total of four bulked groups were obtained, namely, HfR1 (for high ratio of four-seed pod, R1 stage), LfR1 (for low ratio of four-seed pod, R1 stage), HfR3 (for high ratio of four-seed pod, R3 stage), and LfR3 (for low ratio of four-seed pod, R3 stage).

### RNA Extraction, Library Construction, and Sequencing

Total RNA was extracted using RNA purification kit (Tiangen, Beijing, China). The quality and purity of extracted RNA were checked by agarose gel electrophoresis. Oligo(dT) magnetic beads were used to isolated mRNA from total RNA. cDNA libraries were constructed on the Illumina HiSeq sequencing platform with a paired-end read length of 150 bp at Guao Gene Technology (Wuhan, China). FastQC (version 0.11.9) (Brown et al., 2017) was used to evaluate the quality of sequencing data. The raw data were filtered by fastp (version 0.20.0) (Chen et al., 2018) software to remove adapter, unknown bases, and low-quality sequences for obtaining high-quality data.

### Reads Mapping and Differential Expression Analysis

All high-quality clean reads from each bulked group were mapped to the soybean Williams 82 reference genome ver. Wm82.a2.v1 using the HISAT2 (version 2.1.0) software (Kim et al., 2015) with default parameters to obtain the uniquely mapped reads. SAMtools (version 1.9) (Li et al., 2009) was used to convert the mapping SAM files into BAM files and filter the reads with a mapping quality score under 50. The filtered reads were further sorted by chromosomal coordinates. The sorted BAM files were used as input for the htseq-count (version 0.12.4) (Anders et al., 2015) to obtain read counts, which were further used for quantitating the normalized gene expression level as fragments per kilobase of transcript per million mapped reads (FPKM) by the *DESeq2* package (Love et al., 2014). The DEGs in LfR3 versus LfR1, HfR3 versus HfR1, HfR1 versus LfR1, and HfR3 versus LfR3 were identified using the *edgeR* package (Robinson et al., 2010). The genes with the criteria of |log_2_(fold change)| ≥ 1 and false discovery rate (FDR) <0.05 were considered differentially expressed.

### Gene Ontology and Pathway Enrichment Analysis of Differentially Expressed Genes

To characterize the functional categories of the DEGs, gene ontology (GO) was performed on the agriGO v2.0 online website^1^ (Tian et al., 2017). Fisher’s statistical test method followed by FDR (FDR < 0.05) adjustment was used to enrich significant GO terms. To identify the biological pathways of these DEGs, we performed the Kyoto Encyclopedia of Genes and Genomes (KEGG) Orthology Based Annotation System (KOBAS 3.0)^2^ (Xie et al., 2011), which is on the basis of hypergeometric statistical test by comparing the detected genes with the background genes. The pathways with the significance of *p*-value <0.05 were used as the significant enrichment pathways for the input genes that we were concerned about.

### qRT-PCR Analysis

Total RNA was reverse transcribed by StarScript II First-strand cDNA Synthesis Mix With gDNA Remover. Primers were designed using Primer Premier 5, which are listed in Supplementary Table 1. qRT-PCR was performed on the 7500 Software v2.3 with three technical replicates. The relative gene expression levels were calculated using the 2^–ΔΔCt^ method.

## Results

### Phenotypic Characteristics of the Recombinant Inbred Lines

**FIGURE 1:**
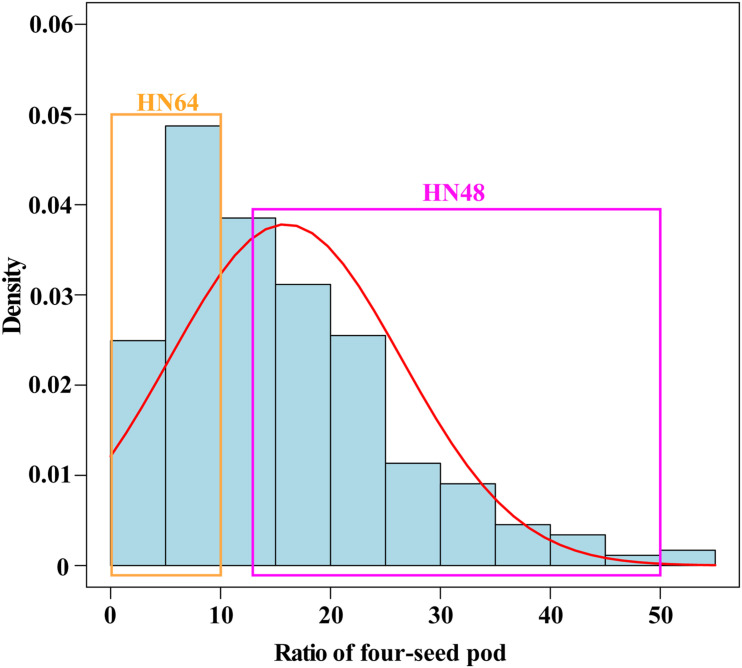
The probability density distribution of the ratio of four-seed pod in the RILs from HN64 × HN48. The selection of lines used for making high ratio of four-seed pod and low ratio of four-seed pod bulked groups for RNA isolation and analysis. RILs, recombinant inbred lines; HN64, Henong 64; HN48, Heinong 48.

To research the genetic mechanism of the ratio of four-seed pod, two soybean cultivars, the variety HN64 with a low ratio of four-seed pods (Supplementary Figure 1A and Figure 1) and the variety HN48 with a high ratio of four-seed pods (Supplementary Figure 1B and Figure 1), were crossed and further inbred for over five generations to construct the RIL population. The flower bud stage (R1 stage) in the RIL was a reproductive stage in soybean when the plants were in the vigorous vegetative growth and began to bloom (Supplementary Figure 1C). The young pod stage (R3 stage) in the RIL was another reproductive stage in soybean when the plants began to pod (Supplementary Figure 1D). The proportion of four-seed pod in the RILs showed a positively skewed distribution (Figure 1 and Supplementary Table 2), which suggests that the trait of the four-seed pod ratio may be affected by multiple loci in this combination.

### Identification of Differentially Expressed Genes by Bulked Segregant RNA Sequencing

**FIGURE 2:**
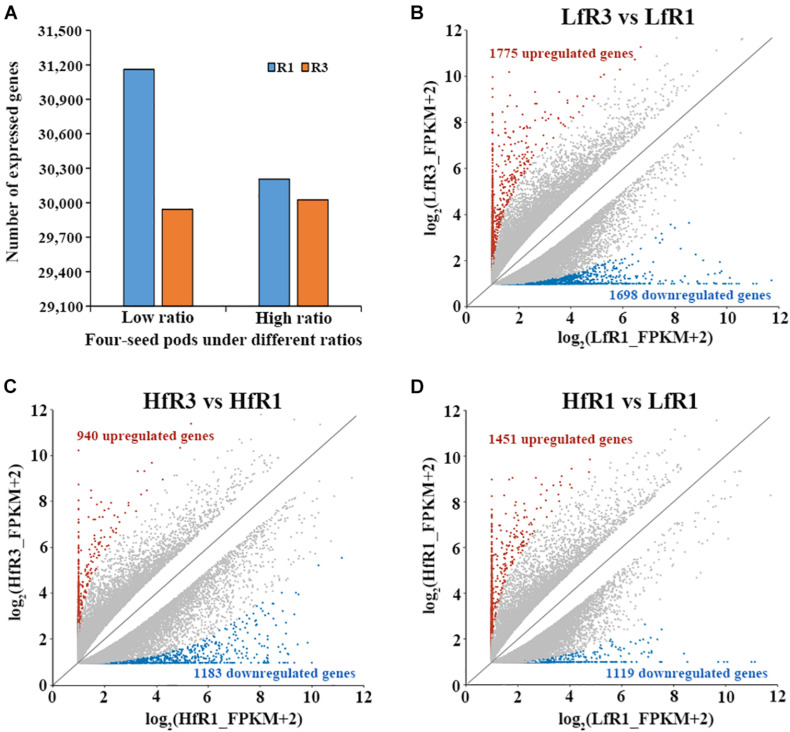
Analysis of gene expression at different samples. **(A)** The number of expressed genes in each sample [fragments per kilobase of transcript per million mapped reads (FPKM) ≥ 1]. **(B–D)** Number of differentially expressed genes in LfR3 versus LfR1, HfR3 versus HfR1, and HfR1 versus LfR1.

To investigate the gene expression pattern during the development of soybean four-seed pods, four transcript libraries were constructed from the tissues of flower buds and young pods. After reads with low quality and adapters were filtered, over 90 million clean reads were generated from the four bulked groups (Supplementary Table 3). Among them, more than 20 million clean reads for each bulked pool were then used for further analysis (Supplementary Table 3). These reads then were mapped to the soybean Williams 82 reference genome ver. Wm82.a2.v1. The percentage of uniquely mapped reads ranged from 51.43 to 86.09% (Supplementary Table 3), and these uniquely mapped reads were further used to calculate the FPKM values. To ensure the confidence of gene expression, we defined genes with FPKM value ≥1 as expressed in at least one of the four bulked groups. The number of expressed genes detected in different bulked groups ranged from 29,943 to 31,160 (Figure 2A).

To detect the DEGs that are related to four-seed pod in the RIL, we compared the bulked groups of two different stages R1 and R3 under the same level of four-seed pod ratio. A total of 3,473 DEGs were detected in LfR3 versus LfR1, of which 1,775 DEGs were upregulated and 1,698 DEGs were downregulated (Figure 2B). Furthermore, 2,123 DEGs were obtained in HfR3 versus HfR1; among them, 940 DEGs were upregulated and 1,183 DEGs were downregulated (Figure 2C). To observe the changes in gene expression at different levels of four-seed pod ratio bulked groups under the same developmental stage, differential expression analysis was performed on HfR1 and LfR1, and HfR3 and LfR3. For HfR1 versus LfR1, 2,570 DEGs were observed, of which 1,451 and 1,119 DEGs were upregulated and downregulated, respectively (Figure 2D). However, only one DEG was identified in the comparison of HfR3 versus LfR3. It was *Glyma.10G089300*, which was significantly upregulated in HfR3 (Supplementary Figure 2). The expression of *Glyma.10G089300* was examined by qRT-PCR using the flower buds and young pods of the HN64 and HN48 parents, respectively, which showed a significantly higher expression level in the young pods of the high four-seed pod ratio parent HN48 (Supplementary Figure 3A). Its homolog in *Arabidopsis* is *AT1G51400*, which encodes a photosystem II 5-kDa protein involved in photosynthesis (Fabro et al., 2008), indicating that it may play a significant role in the development of the number of soybean seeds per pod.

### Gene Ontology and Kyoto Encyclopedia of Genes and Genomes Pathway Enrichment Analysis of Differentially Expressed Genes

**FIGURE 3:**
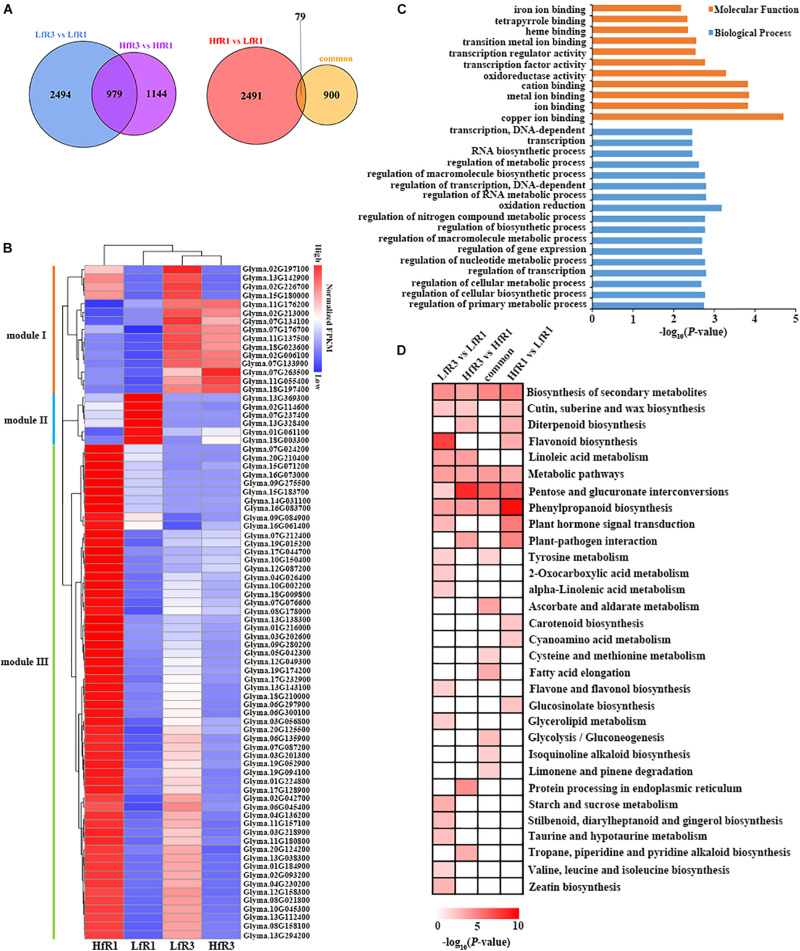
Functional analysis of DEGs. **(A)** Comparison of DEGs between different developmental stages and different ratios of four-seed pod. **(B)** Expression heatmap of 79 overlapped DEGs. **(C)** GO terms of 79 overlapped DEGs (false discovery rate <0.05). **(D)** KEGG enrichment pathways of DEGs. DEGs, differentially expressed genes; GO, gene ontology; KEGG, Kyoto Encyclopedia of Genes and Genomes.

To study the function of DEGs, we focused on the common and specific DEGs at different developmental stages and different levels of four-seed pod ratio. GO analysis was performed on these DEGs. Comparing the DEGs of two different ratios of four-seed pods, a total of 979 common DEGs were identified after LfR3 versus LfR1 was compared with HfR3 versus HfR1 (Figure 3A). These DEGs were significantly enriched in the terms of hydrolase activity, lipid metabolic process, and carboxylesterase activity (Supplementary Figure 4A), which may suggest that these GO terms share a common theme in the development of four-seed pods in two different ratio levels. The number of genes differentially expressed only in the LfR3 bulked group was 2,494 (Figure 3A), which were highly enriched in the chromatin binding, oxidoreductase activity, and hydrolase activity (Supplementary Figure 4B). The only DEGs in the HfR3 bulked group were 1,144 (Figure 3A), and they were associated with enzyme inhibitor activity and external encapsulating structure (Supplementary Figure 4C). The number of DEGs in the high ratio of four-seed pod was less than the low ratio of four-seed pod, which may indicate that gene expression is more stable at a higher ratio of four-seed pod.

Next, we compared the DEGs between HfR1 versus LfR1 and the common DEGs; there were 2,491 DEGs only in HfR1 versus LfR1 (Figure 3A), which mainly enriched in the oxidation reduction, transcription regulator activity, and biosynthetic process (Supplementary Figure 4D). In total, 79 overlapped DEGs were detected between the two sets (Figure 3A). They were divided into three modules according to the clustering of their expression (Figure 3B). Module I contained 15 genes, which showed predominant expression in LfR3 and HfR3. Most of them are laccases. There were six genes in module II, and they were highly expressed in LfR1. The genes in module III were highly expressed in HfR1, and this module contained a total of 58 genes (Supplementary Table 4). All these overlapped DEGs were mainly involved in ion binding, oxidation reduction, transcription regulator activity, and various metabolic processes (Figure 3C).

The KEGG analysis was performed on DEGs to further investigate the enrichment pathways that they were involved in. A total of 31 significantly enriched (*p*-value <0.05) pathways were found. Most DEGs were enriched in biosynthesis of secondary metabolites, metabolic pathways, pentose and glucuronate interconversions, and phenylpropanoid biosynthesis (Figure 3D). Among 79 overlapped DEGs, five DEGs were extremely significantly enriched in plant–pathogen interaction pathway (Supplementary Figure 5). They are four calcium-binding proteins and a WRKY transcription factor (Table 1). Collecting the expression values FPKM of these five genes in different tissues, they showed the highest level of expression in the roots, followed by the nodules^3^ (Supplementary Table 5). These genes may play a crucial role in affecting the ratio of four-seed pods of the aboveground plants through resisting external pathogens and absorbing nutrients by roots.

**TABLE 1:** Annotation of five DEGs enriching in plant–pathogen interaction pathway.

Gene ID	Description	Ortholog	Gene ontology
*Glyma.11G157100*	Putative calcium-binding protein CML19	*AtCML41*	Amino acid import
*Glyma.17G128900*	Putative calcium-binding protein CML19	*AtCML38*	Abscisic acid-mediated signaling pathway; response to fungus
*Glyma.10G002200*	Calmodulin-like protein	*AtCML11*	Peroxisome localization
*Glyma.09G280200*	Probable WRKY transcription factor 33	*AtWRKY33*	Defense response to bacterium and fungus; jasmonic acid-mediated signaling pathway
*Glyma.04G136200*	Putative calcium-binding protein	*AtCML41*	Amino acid import

*DEGs, differentially expressed genes.*

### Genetic Analysis of Differentially Expressed Genes Related to Four-Seed Pod Locus

**FIGURE 4:**
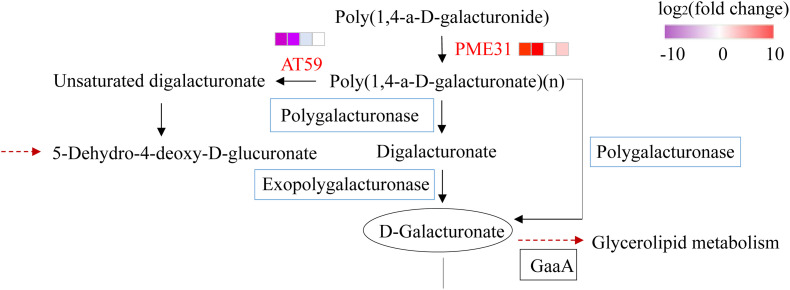
The DEGs located in the QTL interval involved in the pathway of pentose and glucuronate interconversion. Red represents DEGs. Heatmaps represent the expression level. Red represents upregulation, and blue represents downregulation. There were four sets of comparisons, and they were LfR3 versus LfR1, HfR3 versus HfR1, HfR1 versus LfR1, and HfR3 versus LfR3. DEGs, differentially expressed genes; QTL, quantitative trait locus.

To obtain the genes affecting the ratio of four-seed pod, we detected 10 DEGs on chromosome 13 overlapped with the QTL interval associated with four-seed pod, which ranged from 15,306,234 to 16,455,201 (Table 2; Yang et al., 2013). Among them, these genes have their specific roles in the growth and development of plants. *Glyma.13G063100* encodes a mini zinc finger protein 2 that can regulate the development of flower and ovule in tomato (Bollier et al., 2018). *Glyma.13G063800* is a homologous gene with *AT3G28857* in *Arabidopsis*, which encodes a basic helix-loop-helix (bHLH) transcription factor, which can participate in the growth of floral organs (Shin et al., 2019). The KEGG enrichment pathway analysis showed that *Glyma.13G060900* and *Glyma.13G064700* were enriched in the pathway of pentose and glucuronate interconversions, which was related to cell wall modification (Figure 4). In our study, *Glyma.13G060900* was only expressed in LfR3 and HfR3, and it showed a relatively low expression level. However, *Glyma.13G064700* was only expressed in LfR1 and HfR1, and it showed a fairly high expression level (Table 2). qRT-PCR analysis results showed that *Glyma.13G060900* exhibited a low expression level (Supplementary Figure 3B), and *Glyma.13G064700* exhibited a high expression level in all the samples (Supplementary Figure 3C). *Glyma.13G060900* encodes pectin methylesterase (PME) 31, which is a homologous gene with *Arabidopsis AT3G29090* (*AtPME31*), and it was highly expressed in silique (Louvet et al., 2006). *Glyma.13G064700* encoding pectate lyase is homologous to *Arabidopsis AT1G14420* (*AtAT59*) (Sun and van Nocker, 2010). Observing their expression changes at different developmental stages and different ratios of four-seed pod, we found that *Glyma.13G060900* was upregulated at two different levels of R3 stages and in HfR3 versus LfR3, and *Glyma.13G064700* was downregulated at two different levels of R3 stages and in HfR1 versus LfR1 (Figure 4). These two genes specially expressed at the flower bud stage and the young pod stage. Collecting public data from Phytozome database,^3^ we found that the expression of *Glyma.13G060900* in pods is higher than that of other tissues, except for the leaves and unopened flowers, and *Glyma.13G064700* only shows a specific high expression in flowers (Supplementary Table 6). These results are almost consistent with those of the article, which showed that they may play unique roles in the different developmental stages of the four-seed pod. They were considered to be main candidates for further molecular research on affecting the ratios of four-seed pods in soybean.

**TABLE 2:** Ten DEGs on chromosome 13 overlapped with the QTL interval associated with the trait of four-seed pod.

Gene ID	Start	End	FPKM				Description
			LfR1	LfR3	HfR1	HfR3	
*Glyma.13G055600*	15,309,709	15,313,747	2.76	0.03	0.40	0.75	Haloacid dehalogenase-like hydrolase (HAD) superfamily protein
*Glyma.13G059700*	15,703,489	15,706,171	6.43	15.08	1.27	44.49	Early nodulin-like protein 20
*Glyma.13G060900*	15,841,355	15,845,079	0.00	1.51	0.00	3.37	Pectin methylesterase 31
*Glyma.13G061300*	15,859,700	15,860,150	3.26	0.00	1.35	0.00	NA
*Glyma.13G062100*	15,978,935	15,979,881	0.00	0.20	2.31	0.00	Transmembrane protein
*Glyma.13G063000*	16,109,728	16,111,701	0.00	0.12	1.17	2.17	NA
*Glyma.13G063100*	16,117,664	16,118,872	0.00	692.15	99.45	705.85	Mini zinc finger 2
*Glyma.13G063800*	16,302,368	16,304,936	2.68	4.54	0.00	5.07	Basic helix-loop-helix (bHLH) DNA-binding family protein
*Glyma.13G064700*	16,424,596	16,427,361	1,153.81	0.00	291.60	0.00	Pectate lyase family protein
*Glyma.13G065100*	16,450,914	16,453,876	0.00	0.00	3.11	0.00	NA

*DEGs, differentially expressed genes; QTL, quantitative trait locus; FPKM, fragments per kilobase of transcript per million mapped reads.*

## Discussion

As a potential factor affecting yield, the ratio of four-seed pod is not only regulated by genes but also affected by the external environment and the internal developmental stages. Recent genetic studies have identified *Ln* gene and many main effect QTLs that affect the trait of the four-seed pod, but it is essential to further study the main effect genes affecting the ratios of four-seed pod. In this study, BSR-seq was performed on the RIL progenies constructed by two elite varieties from the northeast of an important soybean producing area, cultivar HN48 has more four-seed pods, and cultivar HN64 has less four-seed pods. Combined with gene differential expression analysis and pathway enrichment analysis, eight candidate genes and their regulatory pathways were preliminarily obtained. Early genetic studies have identified an *Ln* gene, and it can affect the number of four-seed pods (Jeong et al., 2012; Fang et al., 2013). *Ln* gene affected the formation of four-seed pod by controlling the division and expansion of early flowering cells (Fang et al., 2013; Sayama et al., 2017). The early flowering stage is the key time point for the formation of four-seed pod. In our study, the expression of *Ln* gene (*Glyma.20G250000*) did not show significant difference at the flower bud stage versus the young pod stage and under two different ratios of four-seed pod, and its expression was relatively stable at different developmental stages under the same four-seed pod level (Supplementary Figure 6). The qRT-PCR verification results showed that, except for the young pod of HN48, *Ln* gene was stably expressed in other samples (Supplementary Figure 3D). We speculated that there might be other genes involved in regulating the ratios of four-seed pod in our RIL population, which may be specific environmental conditions that trigger genes to participate in the corresponding regulatory pathways. This is likely the phenotypic effect of crop traits brought by the interaction of environment and genes. The RIL population we constructed was only from the single environment of Beijing. The distribution of phenotypic data showed that the trait of four-seed pod was controlled by multiple loci; some of them may be minor loci. These minor loci often contain environmentally sensitive genes, and these genes are likely to affect the ratio of four-seed pod by activating corresponding regulatory pathways.

The formation of the number of soybean seeds per pod is not only controlled by *Ln* gene but also affected by the signaling pathways activated by genes. In this study, two important developmental stages of soybean, namely, the flower bud stage and the young pod stage, were used for sampling. The young pod stage is the initial period of the formation of the number of soybean seeds per pod. We identified the only DEG *Glyma.10G089300* in HfR3 versus LfR3, which encodes a photosystem II 5-kDa protein and is an important protein involved in photosynthesis. The expression level of this gene in HfR3 was significantly higher than that in LfR3, and the difference was about seven times. It is speculated that stronger photosynthesis occurred in HfR3 and more photosynthetic products were accumulated, which promoted the formation of a higher ratio of four-seed pod to some extent. Here, we identified 79 genes that were differentially expressed at different developmental stages and at different ratios of four-seed pod. Only the pathway of plant–pathogen interaction was where they were significantly enriched, and five DEGs were involved in the regulation of this pathway. They encode four calcium-binding proteins and a WRKY transcription factor. Previous studies have shown that calcium plays a critical role in the formation of plant cell wall (Langer et al., 2019). Complete and rigid cell walls help plants resist the invasion of pathogens from the outside (Hepler, 2005). Preventing the interference of pathogens from the outside is the prerequisite for healthy growth of plants. Moreover, calcium can promote the growth of root hairs (Zhang et al., 2021). In our study, these calcium-binding genes showed high expression characteristics in roots, indicating that plants with vigorous roots could absorb more nutrients and thus contribute to the accumulation of nutrients by the aboveground leaves, flowers, and pods. This may be a key factor affecting the ratio of four-seed pods, which is different from the mechanism of *Ln* gene on four-seed pod. In the study of rice grain shape, a calmodulin-binding gene was identified in the target interval by bulked-segregant analysis (BSA) sequencing, which has shown to possess the potential to regulate rice yield (Wu et al., 2020). As a key component of pathogen defense and plant development, WRKY transcription factor can participate in ABA-mediated signaling pathway (Rushton et al., 2012). ABA secreted from soybean roots may control the expansion of pods. It is indicated that WRKY transcription factor may be involved in the regulation of the four-seed pod formation. We identified 10 DEGs in the reported QTL interval of four-seed pod, and their enrichment pathway was pentose and glucuronate interconversions. There were two DEGs involved in this pathway encoding PME and pectate lyase. Study on silique development in *Arabidopsis* has shown that *AtPME31* is a member of the PME gene family and does not possess a PRO region. Based on the expression diversity of PME family genes in silique development, it is speculated that they are involved in regulating pectin modification network during fruit ripening (Roberts, 2000; Louvet et al., 2006). *AT59* is one of the members of the pectate lyase-like (*PLL*) gene family and can catalyze depolymerizing pectin. Related study has shown that it was specifically expressed in the pollen and stamen of *Arabidopsis thaliana* (Sun and van Nocker, 2010). This was consistent with the result that *AT59* was only expressed in floral bud in our study. It can be seen that the two genes play a vital role in the metabolism and transformation of sugars, which was essential for sugar accumulation related to the formation of the number of seeds per pod. The DEGs identified in this single environment of Beijing coincided with the previously mapped QTL in multiple environments, indicating that the genes at this locus have a certain degree of conservation. They may be the key node genes in the pathways regulating the ratios of four-seed pod.

**FIGURE 5:**
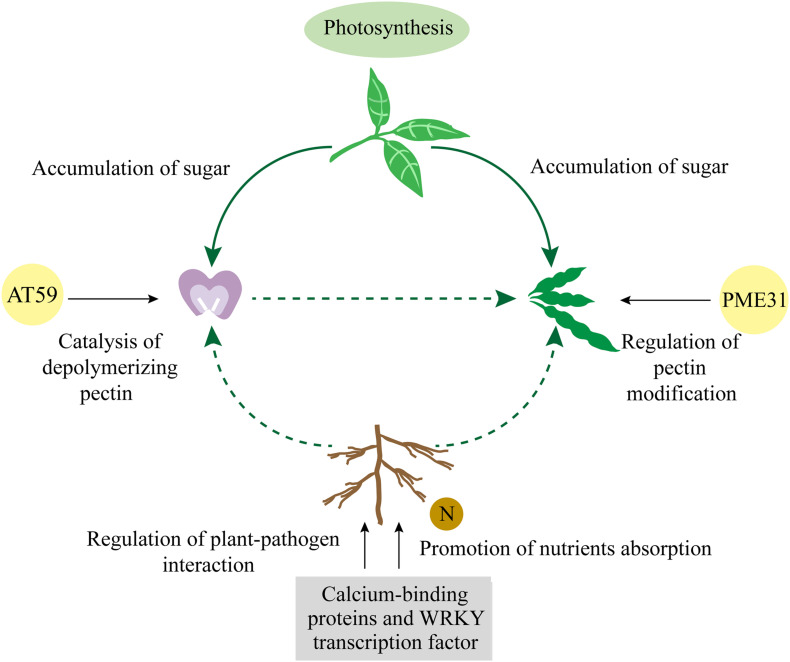
The comprehensive model for DEG regulating pathways affecting the ratios of four-seed pod in soybean. DEGs, differentially expressed genes.

Given these results together, we propose a model based on gene regulation pathways under specific environment that affects the ratios of four-seed pod through the accumulation of nutrients (Figure 5). Under certain environment, aboveground plants promoted photosynthetic efficiency of leaves by increasing the expression of important genes in the photosystem and further accumulated more photosynthetic products to improve the ratio of four-seed pod. Underground plants mainly depended on the resistance of roots to external pathogens and their absorption of nutrients to affect the ratio of four-seed pod above the ground. The expression of calcium-binding genes in soybean nodules was observed to be ranked only second to roots. It is possible that these genes play an important role in the nitrogen fixation and nodulation in roots, which is conducive to the absorption of nutrients by roots. Therefore, whether nitrogen fixation and nodulation in soybean roots affect the number of seeds per pod in the aboveground plants is the focus of our further study. Soybean shell, as a direct protective barrier for the grains, provides a large amount of nutrients for the growth of grains. Studies in peanuts have shown that calcium first affects the development of the shell and the absorption of nutrients (Yang et al., 2020). The nutritional status of soybean shell may play a vital role in the formation of the number of seeds per pod. So it is worthy of further consideration to study the mechanism of calcium in the formation of soybean four-seed pod. As the main component of plant cell walls, pectin is a class of polysaccharide polymer. It plays an important role in the morphogenesis of fruits and the defense of pathogenic bacteria (Mohnen, 2008; Bethke and Glazebrook, 2019). It laid the cytological foundation for the formation of the number of seeds per pod. The results of this study indicated that pectin had different mechanisms of action during different developmental stages of soybean pods. At the floral bud stage, polysaccharide polymers were depolymerized into small molecular sugars. At the young pod stage, pectin was mostly polymerized, and demethylesterification at this time may improve the firmness of cell wall. Therefore, further research on the pectin metabolism pathway and key node genes at reproductive developmental stage of soybean have a significant value to improve the ratio of four-seed pod.

## Data Availability Statement

We have uploaded the RNA sequencing data to the repository SRA. The bioproject ID is PRJNA735557.

## Author Contributions

LS designed the research. YB collected the samples. TF and WH performed the research. TF wrote the article with input from LS. YW, ZY, XLu, and XLi performed the RIL population construction. All authors contributed to the final manuscript revision.

## Conflict of Interest

The authors declare that the research was conducted in the absence of any commercial or financial relationships that could be construed as a potential conflict of interest.

## Publisher’s Note

All claims expressed in this article are solely those of the authors and do not necessarily represent those of their affiliated organizations, or those of the publisher, the editors and the reviewers. Any product that may be evaluated in this article, or claim that may be made by its manufacturer, is not guaranteed or endorsed by the publisher.

## Footnotes

1
http://systemsbiology.cau.edu.cn/agriGOv2/

2
http://kobas.cbi.pku.edu.cn/kobas3/

3
https://phytozome.jgi.doe.gov/

## Supplementary Material

Supplementary Figure 1 The phenotypes of the parental varieties HN64 **(A)** and HN48 **(B)**. Plant materials of the flower bud (R1 stage) **(C)** and the young pod (R3 stage) **(D)**.

Supplementary Figure 2 The expression level of *Glyma.10G089300* in LfR1, LfR3, HfR1, and HfR3 four bulked groups.

Supplementary Figure 3 Expression of *Glyma.10G089300* **(A)**, *Glyma.13G060900* **(B)**, *Glyma.13G064700* **(C)**, and *Glyma.20G250000* **(D)** in flower bud and young pod of the high four-seed pod ratio parent HN48 and the low four-seed pod ratio parent HN64 were detected by qRT-PCR. The expression level of each gene was normalized to an *Actin* gene. Expression levels are shown as mean ± SE from three replicates.

Supplementary Figure 4 Gene ontology terms analysis of DEGs respectively in panel **(A)** the overlapped DEGs of LfR3 versus LfR1 and HfR3 versus HfR1, **(B)** the only DEGs of LfR3 versus LfR1 compared with HfR3 versus HfR1, **(C)** the only DEGs of HfR3 versus HfR1 compared with LfR3 versus LfR1, **(D)** the only DEGs of HfR1 versus LfR1 compared with the common DEGs.

Supplementary Figure 5 Plant-pathogen interaction pathway enriched from the 79 overlapped DEGs.

Supplementary Figure 6 The changes in expression level of *Glyma.20G250000* in LfR1, LfR3, HfR1, and HfR3 four samples.

Supplementary Table 1 The primers used in qRT-PCR.

Supplementary Table 2 The analysis of phenotypic data for four-seed pod of the RILs population.

Supplementary Table 3 Statistics of bulked segregant RNA-seq reads.

Supplementary Table 4 Expression and annotation information of 79 overlapped DEGs.

Supplementary Table 5 FPKM values of five genes in multiple tissues.

Supplementary Table 6 FPKM values of two tissue-specific expression genes in multiple tissues.
