# Supplementary figures and images for: Identification of Potential Gene Regulatory Pathways Affecting the Ratio of Four-Seed Pod in Soybean
Source: Front Genet. 2021 Sep 1;12:717770. doi: 10.3389/fgene.2021.717770 (PMC8440838; doi:10.3389/fgene.2021.717770)

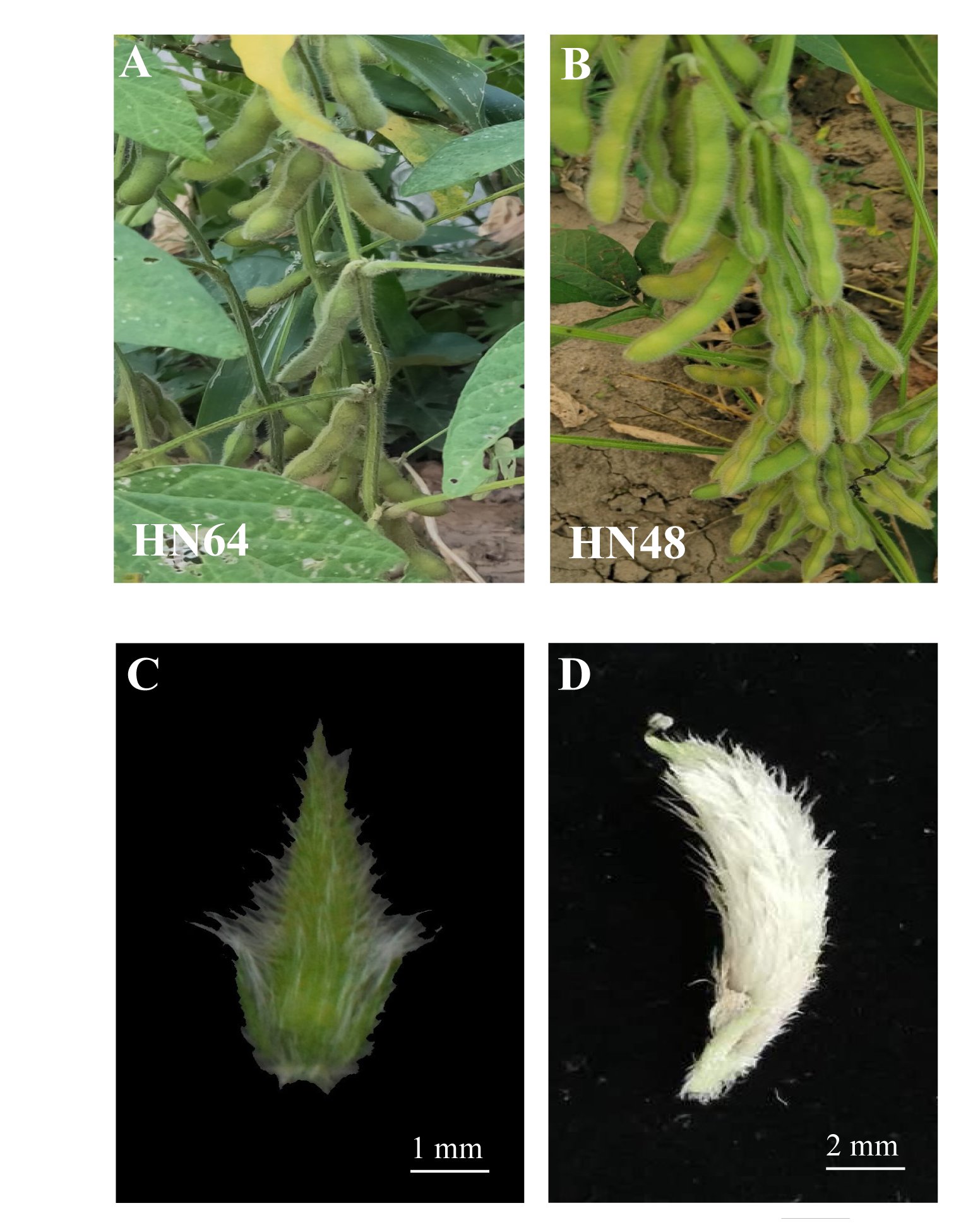

Supplement: Supplementary Figure 1 — The phenotypes of the parental varieties HN64 (A) and HN48 (B). Plant materials of the flower bud (R1 stage) (C) and the young pod (R3 stage) (D). [file Image_1.TIF]

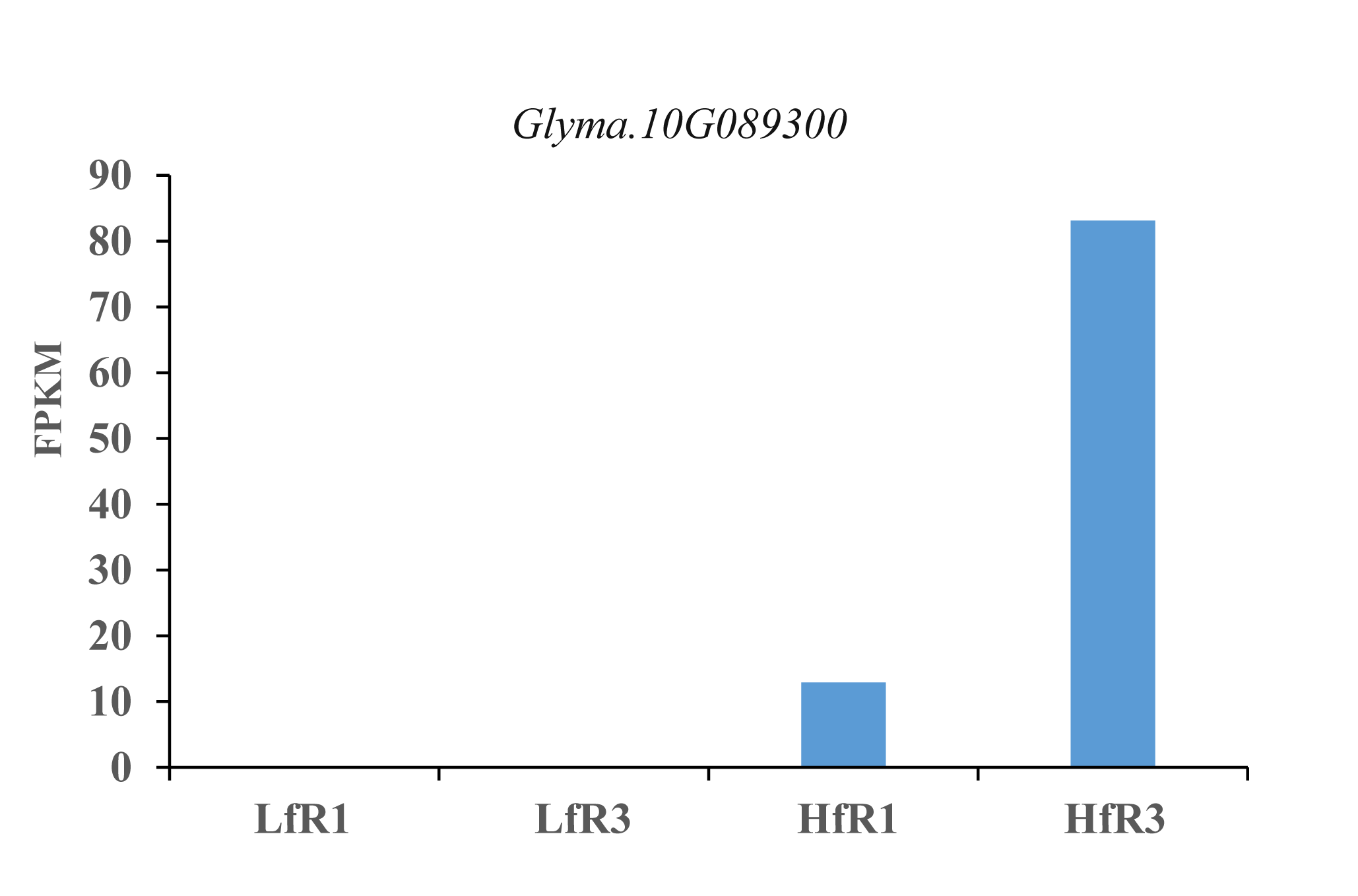

Supplement: Supplementary Figure 2 — The expression level of Glyma.10G089300 in LfR1, LfR3, HfR1, and HfR3 four bulked groups. [file Image_2.TIF]

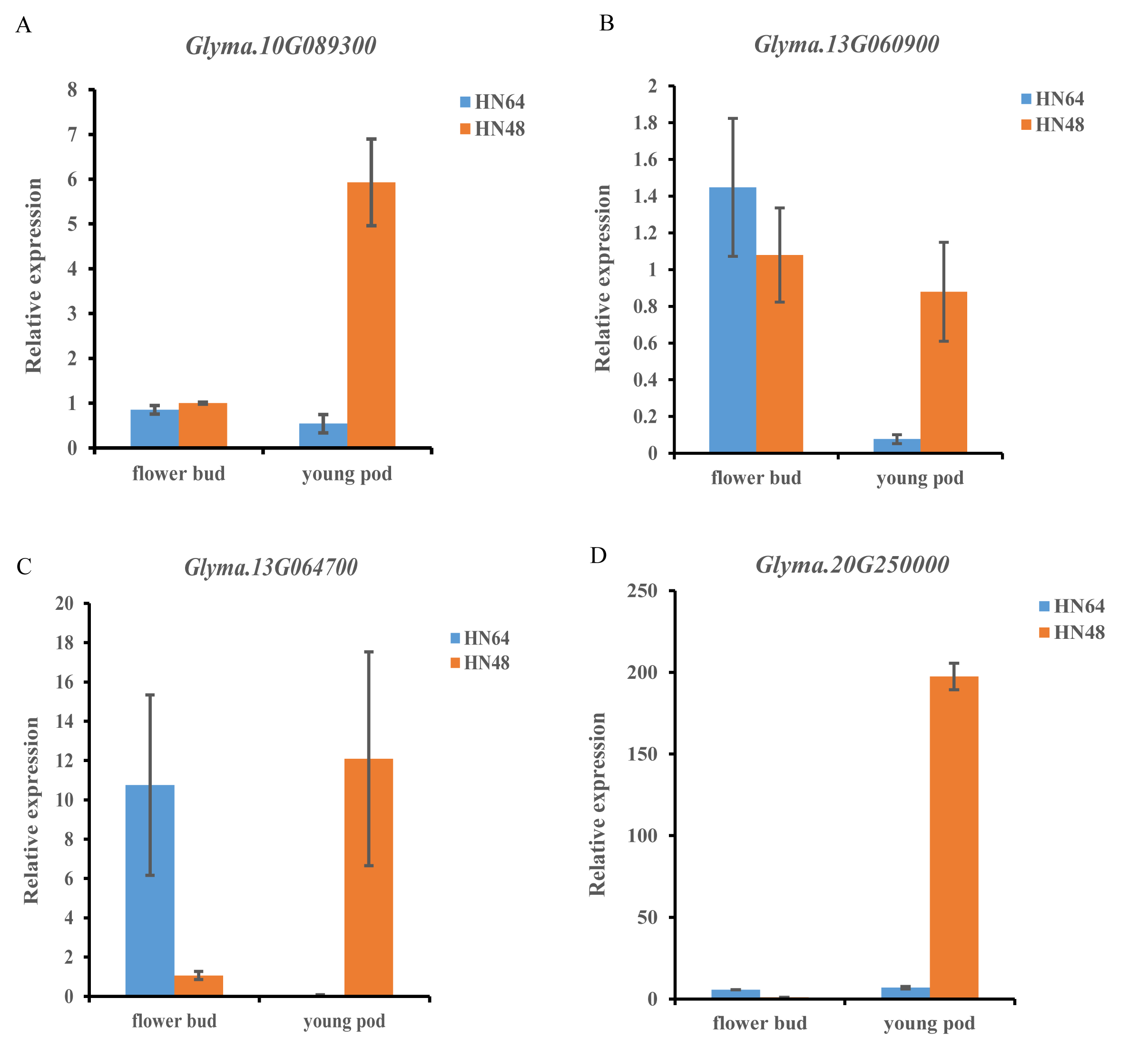

Supplement: Supplementary Figure 3 — Expression of Glyma.10G089300 (A), Glyma.13G060900 (B), Glyma.13G064700 (C), and Glyma.20G250000 (D) in flower bud and young pod of the high four-seed pod ratio parent HN48 and the low four-seed pod ratio parent HN64 were detected by qRT-PCR. The expression level of each gene was normalized to an Actin gene. Expression levels are shown as mean ± SE from three replicates. [file Image_3.TIF]

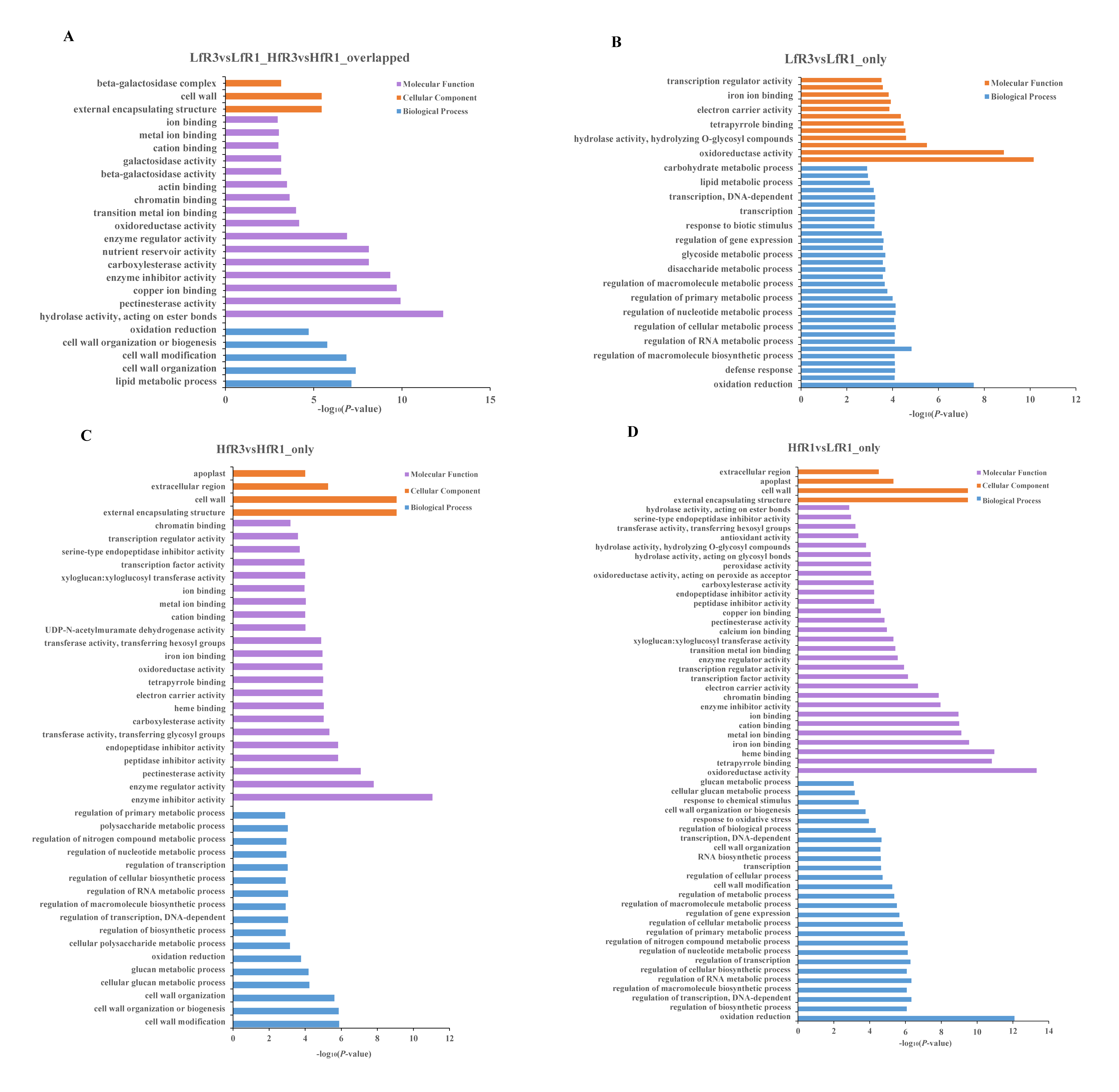

Supplement: Supplementary Figure 4 — Gene ontology terms analysis of DEGs respectively in panel (A) the overlapped DEGs of LfR3 versus LfR1 and HfR3 versus HfR1, (B) the only DEGs of LfR3 versus LfR1 compared with HfR3 versus HfR1, (C) the only DEGs of HfR3 versus HfR1 compared with LfR3 versus LfR1, (D) the only DEGs of HfR1 versus LfR1 compared with the common DEGs. [file Image_4.TIF]

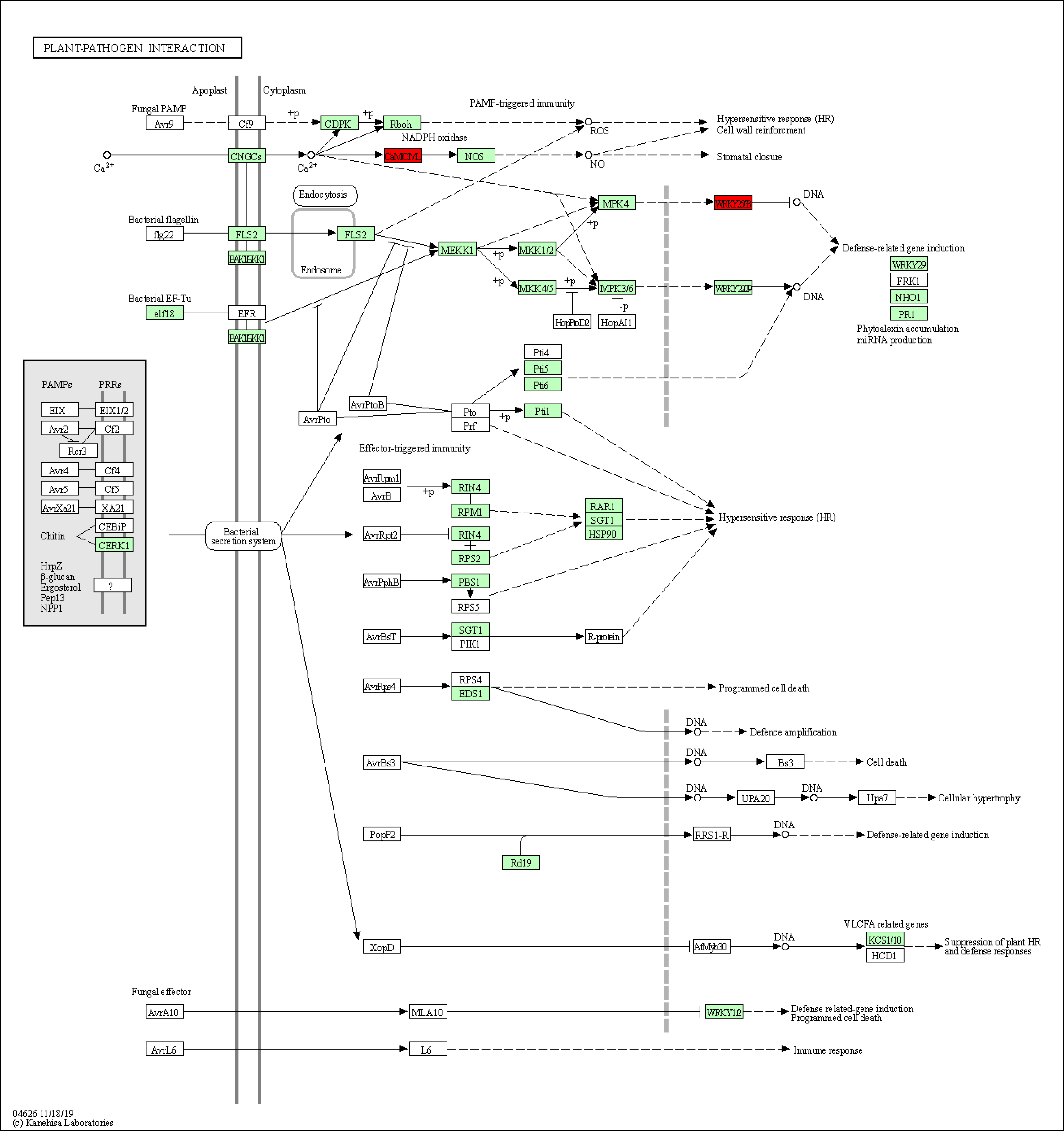

Supplement: Supplementary Figure 5 — Plant-pathogen interaction pathway enriched from the 79 overlapped DEGs. [file Image_5.TIF]

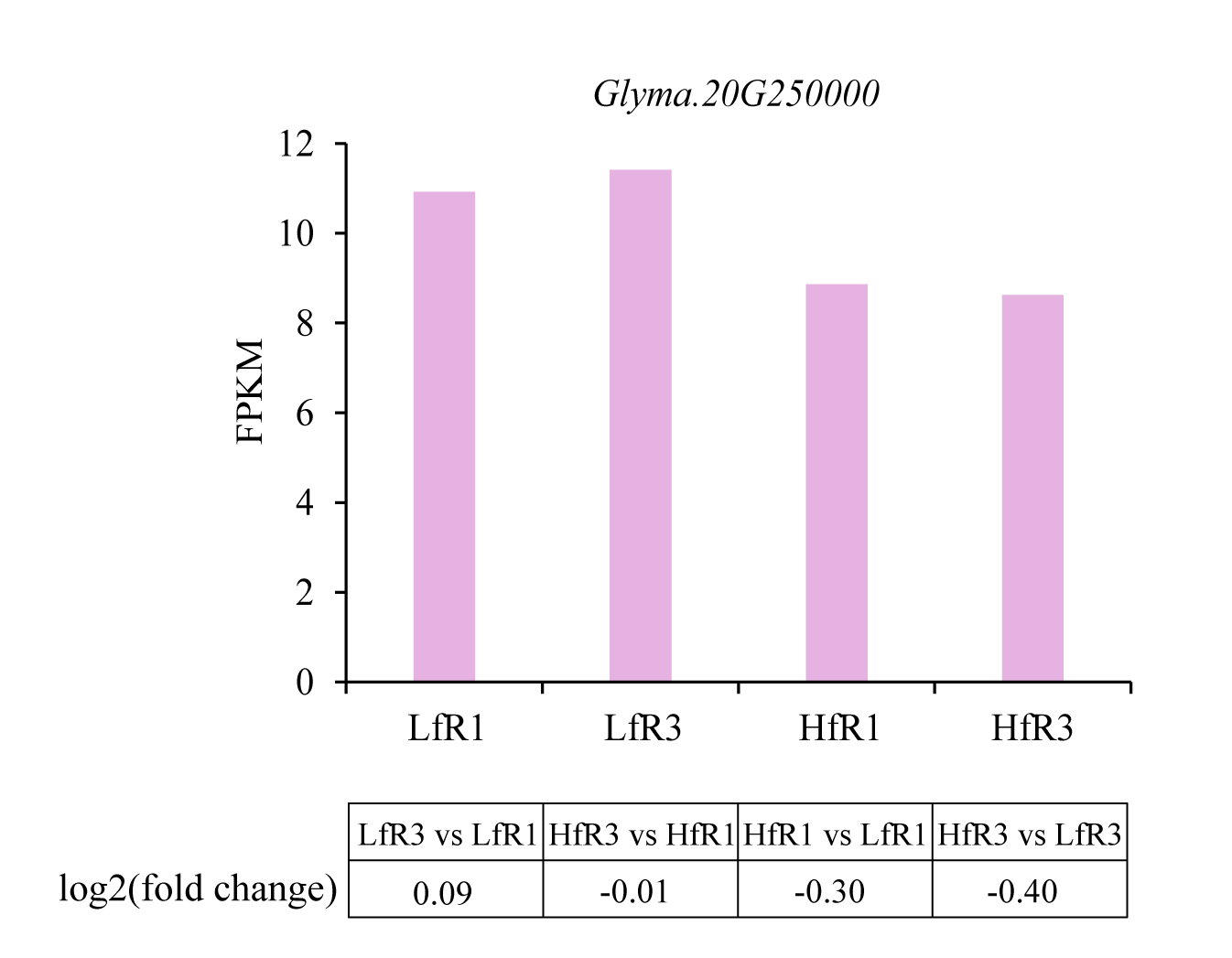

Supplement: Supplementary Figure 6 — The changes in expression level of Glyma.20G250000 in LfR1, LfR3, HfR1, and HfR3 four samples. [file Image_6.TIF]
